# Preserved social facilitation and preferential processing of partner-relevant information in Huntington’s disease

**DOI:** 10.1093/braincomms/fcae462

**Published:** 2024-12-19

**Authors:** Jan Van den Stock, Gert Cypers, François-Laurent De Winter, Mathieu Vandenbulcke

**Affiliations:** Center Neuropsychiatry, Department of Neurosciences, Psychiatry Research Group, Leuven Brain Institute, KU Leuven, 3000 Leuven, Belgium; Geriatric Psychiatry, University Psychiatric Center KU Leuven, 3000 Leuven, Belgium; Department of Neurology, Onze-Lieve-Vrouw Hospital, 9300 Aalst, Belgium; Department of Neurology, Onze-Lieve-Vrouw Hospital, 9300 Aalst, Belgium; Center Neuropsychiatry, Department of Neurosciences, Psychiatry Research Group, Leuven Brain Institute, KU Leuven, 3000 Leuven, Belgium; Geriatric Psychiatry, University Psychiatric Center KU Leuven, 3000 Leuven, Belgium; Center Neuropsychiatry, Department of Neurosciences, Psychiatry Research Group, Leuven Brain Institute, KU Leuven, 3000 Leuven, Belgium; Geriatric Psychiatry, University Psychiatric Center KU Leuven, 3000 Leuven, Belgium

## Abstract

This scientific commentary refers to ‘The joint memory effect: challenging the selfish stigma in Huntington’s disease?’, by Dalléry *et al*. (https://doi.org/10.1093/braincomms/fcae440).

**This scientific commentary refers to ‘The joint memory effect: challenging the selfish stigma in Huntington’s disease?’, by Dalléry *et al*. (https://doi.org/10.1093/braincomms/fcae440).**

Huntington’s disease is a neurodegenerative disorder that may affect social cognition as the disease progresses, for instance regarding emotion recognition, mentalizing or empathy.^1^ These alterations have typically been interpreted primarily as a direct effect of the disease-related structural and functional brain changes.^2^ However, they may have a multifactorial aetiology. The psychological stressors associated with the knowledge of carrying a hereditary pathogenic mutation and experiencing the emergence or progression of motor, cognitive or psychiatric symptoms may affect social behaviour. Furthermore, the reaction of others to symptom manifestation may have an impact on social cognition. Of note, social cognition in neurodegenerative disorders like Huntington’s disease has typically been investigated using psychophysical tasks such as forced choice emotion categorization of pictures displaying emotional expressions or self-assessment questionnaires.^3,4^ This approach often has limited ecological validity, relying substantially on artificial stimuli that may not entirely capture the complexity and subtleties of real-life social interactions. These tasks may also partly depend on language, memory and attentional abilities, making it difficult to isolate specific social cognitive functions from broader cognitive abilities that may be impacted in these disorders. Furthermore, the reductionist nature of these tasks limits their sensitivity to subtle, context-dependent social characteristics, which are often typical changes, particularly in the early stages of neurodegenerative diseases.

Dalléry *et al*.^5^ advance the state-of-the-art and explore the prevailing belief that Huntington’s disease initiates a disregard for the presence of others and the processing of their mental states. To address this issue, the authors leverage the Joint Memory Paradigm, which measures the ability to encode, store and retrieve information in both a social and non-social context, as well as the tendency to preferentially process information that is only directly relevant to others. In particular, they presented participants with Huntington’s disease and healthy controls with words from three semantic categories during a semantic detection task. In the ‘Pair’ condition, the participant and an examiner acting as a partner were each assigned one category to respond to, while the third category remained unassigned. In the ‘Alone’ condition, the participants performed the same task individually. After categorization, participants underwent an incidental free-recall task where they reproduced as many words as possible. The key social cognitive parameters relate to the proportion of reproduced words assigned to themselves, their partner and the unassigned category, as well as the effect of the presence of a partner.

Here, we further discuss two major converging findings that underscore the presence of implicit social cognitive abilities in Huntington’s disease. The first one relates to the long-known so-called ‘social facilitation’ effect that refers to a performance increase in a social compared to a non-social context.^6^ Dalléry’s results reveal a social facilitation effect in Huntington’s disease, particularly for self-relevant words. The presence of another person thus introduces a social dynamic that results in a better encoding, possibly through heightening the participant’s motivation to perform well. This socially induced effect may amplify the focus on self- and partner-relevant information. Importantly, the social facilitation of the self-relevant words was proportional between control and Huntington’s disease groups. Self-prioritization is closely linked to self-referential processing, referring to a deeper and more efficient processing of self-relevant information. In the Pair condition, this effect may be heightened as the participant contrasts self-relevant information with their partner’s assigned items. The distinction between self and other may become more salient, leading to an increased focus on the self-relevant category. The presence of a partner may also trigger social comparison processes where individuals evaluate their performance relative to others. In the Pair condition, participants may thus focus more on their own assigned category to ensure that they are contributing adequately or out-performing their partner. This comparative aspect may drive them to prioritize self-relevant information more strongly compared to when they are alone, where such comparisons are absent. Thus, the social facilitation for self-relevant information may arise from heightened social motivation, stronger self-referential processing and social comparison mechanisms, all of which lead participants to prioritize self-relevant information when engaging in a collaborative task. Importantly, this appears preserved in Huntington’s disease.

Second, the Joint Memory Paradigm allows assessment of whether individuals spontaneously and implicitly prioritize partner-related information, compared to irrelevant information. The results of Dalléry and colleagues revealed that in the Huntington’s disease group, the proportion of reproduced words from the partner’s category was significantly higher than the proportion of reproduced words from the unassigned category. With this, the authors provide evidence for the presence of the so-called ‘Joint-Memory Effect’ (JME) in Huntington’s disease. JME is defined as higher rates of recall for the partner’s target words compared to irrelevant words.^7^ The JME taps into several key aspects of social cognition, particularly mentalizing, attention to social cues and collaborative memory processes. The central aspect of social cognition reflected by the JME is the capacity for shared attention and implicit collaborative encoding, i.e. the ability to attend to and prioritize information that is only directly relevant to an assigned partner. In the study of Dalléry *et al*., the Alone condition was always followed by the Pair condition and there were no partner-relevant words for the participant in the Alone condition. Future studies may further explore whether the JME persists in Huntington’s disease when the partner is not physically present, e.g. by letting the Pair condition precede the Alone condition or adapting the instructions in the Alone condition to establish a category only relevant for others.

At the neural level, the findings of Dalléry *et al*. provide limited significant and biologically plausible robust structural associations, i.e. a negative correlation between JME and grey matter volume of a region in the right orbitofrontal cortex. However, the relevant brain–behaviour correlations may be primarily detectable at the functional level. In healthy populations, JME-like paradigms have typically been associated with regions of the mentalizing network, such as the medial prefrontal cortex.^8^ However, this region is strongly connected to the striatum, which is affected early in Huntington’s disease. The medial prefrontal cortex may also be structurally affected as Huntington’s disease progresses. However, structural neurodegeneration may induce compensatory functional mechanisms. This may consist of functional recruitment of regions with more optimal structural integrity that may help maintain cognitive or behavioural functional capacity.^9^

The findings from Dalléry *et al*. provide a more positive outlook for Huntington’s disease by challenging the notion that social cognitive abilities are uniformly impaired in individuals with the disease. While Huntington’s disease may affect various aspects of cognition, including social cognition, the study demonstrates that some social cognitive mechanisms remain intact, offering hope for preserving and potentially improving quality of life for those with Huntington’s disease. First, the preservation of the social facilitation effect in Huntington’s disease is a promising indication and suggests that the social environment plays a significant role in bolstering cognitive function, specifically in tasks involving self-relevant information. The fact that Huntington’s disease participants benefit from the presence of a partner indicates that social dynamics can still positively influence cognitive performance. This insight is crucial because it highlights the potential for social interventions, such as group activities or therapies that emphasize interaction with others, to maintain or even enhance certain cognitive functions in Huntington’s disease. Rather than viewing social cognitive deficits as irreversible and widespread, these findings suggest that the right social context can provide cognitive support for individuals with Huntington’s disease.

Moreover, the demonstration of the JME in Huntington’s disease patients offers another optimistic view of social cognition. The ability to prioritize partner-relevant information implicitly, as shown in the study, suggests that even in the presence of neurodegeneration, individuals with Huntington’s disease retain an ability to engage in shared attention and collaboration. While the effect was less pronounced compared to the control group, it was nonetheless present, indicating that individuals with Huntington’s disease are still attuned to the needs and perspectives of others, even when they do not belong to the social inner realm. This retention of collaborative memory processes speaks to the resilience of certain social cognitive functions in Huntington’s disease, which may be leveraged to develop supportive environments where these abilities can be utilized and maintained. Rather than assuming a blanket decline in all aspects of social functioning, it highlights the areas of resilience and potential for intervention, similar to recent initiatives in other neurodegenerative disorders.^10^

## Data Availability

No new data were generated or analysed in support of this research.
